# The relationship between triglyceride, cholesterol and lipoprotein levels, and immune responses to hepatitis B vaccine

**DOI:** 10.3389/fmed.2023.1131373

**Published:** 2023-03-30

**Authors:** Dan Guo, Jiazhen Dai, Rong Ju, Qifan Zhou, Nenghuan Wang, Chunhua Wu, Hui Tao, Hui Jing, Chen Zhu, Jinxian Mao, Jiayan Xu

**Affiliations:** ^1^Department of Preventive Health, The Affiliated Jiangning Hospital of Nanjing Medical University, Nanjing, China; ^2^Department of Gynaecology and Obstetrics, The Affiliated Jiangning Hospital of Nanjing Medical University, Nanjing, China

**Keywords:** hepatitis B vaccine, triglyceride (TG), cholesterol, high-density lipoprotein (HDL), low-density lipoprotein (LDL), immune response

## Abstract

Cholesterol homeostasis disorder and hypertriglyceridemia, as common metabolic conditions, have rarely been reported to affect the immune responses to the hepatitis B vaccine. Our study found that higher high-density lipoprotein (HDL) level showed a significant relationship with positive anti-HBs results (cOR = 1.479, 95% CI: 1.150, 1.901, *p* = 0.002; aOR = 1.304, 95% CI: 1.006, 1.691, *p* = 0.045), especially in individuals aged 18- to 40-year-old, female, smoking more than 100 cigarettes in life, and drinking more than 12 times every year. Lower low-density lipoprotein (LDL) level was associated with a negative anti-HBs result among participants aged 18- to 40-year-old, and participants who were obese. Higher level of HDL and lower level of LDL may be protective factors of better immune effect of hepatitis B vaccine. More research should be conducted to investigate the influence of the cholesterol level on the immune responses to the hepatitis B vaccine, and more in-depth research should be performed to uncover the mechanism.

## 1. Introduction

Hepatitis B, caused by the hepatitis B virus (HBV), is a group of systemic infectious diseases that may lead to chronic liver diseases, including cirrhosis, liver necrosis, and liver cancer. HBV infection is an important worldwide public health problem and is the crucial cause of hepatocellular carcinoma (HCC), which causes significant morbidity and mortality worldwide. Globally, an estimated 887,220 people died from HBV infection, including acute hepatitis, cirrhosis and HCC, in 2015 according to the World Health Organization (WHO). Clearly, HBV infection poses an enormous economic and social burden.

Undoubtedly, compared to other interventions, vaccines are the most cost-effective and safe defense against infectious diseases. Likewise, the best defense against HBV infection, both in terms of benefit–cost ratios and cost-effectiveness, is hepatitis B immunization (1). Mathematical modeling has shown that an estimated 210 million new HBV infections globally have been prevented since the implementation of worldwide hepatitis B vaccination programs (2). HBV carrier rates and chronic complications of HBV infection have markedly declined in countries that have adopted vaccine programs based on WHO recommendations (3, 4).

People without effective immunity are susceptible to HBV infection. Studies have shown that many factors influence the seroprotective response to hepatitis B vaccination. Age is an important factor; notably, the older the age of the patient (more than 40 years old) is, the lower the immune response to the hepatitis B vaccine (5). In addition, smoking, male sex, chronic disease, and infectious diseases such as HIV infection also cause a lower response. Furthermore, the role of genetic factors, as well as epigenetic factors, in vaccine response cannot be ignored. In addition, evidence suggests that obese individuals are less likely to achieve a seroprotective response to hepatitis B vaccination, but they have higher susceptibility to bacterial, viral and fungal infections (6–9). Triglyceride (TG) and cholesterol are important lipid molecules in mammals. Hypertriglyceridemia, with a parallel prevalence of mild-to-moderate to obesity, is a common clinical health problem (10). It has been established that hypertriglyceridemia is a crucial contributor to pancreatitis as well as atherosclerotic cardiovascular disease (ASCVD). Cholesterol is an essential structural component of eukaryotic cell membranes and determines the physiological properties of cell membranes. Its metabolic disorders, both excess and deficiency, are associated with cardiovascular and cerebrovascular diseases, diabetes, cancers and other diseases (11, 12). Besides, cholesterol plays a key role in regulating immune function, which is demonstrated to be very sensitive to the variations of this macronutrient concentration (13). High-density lipoprotein (HDL) may play critical roles both in innate and adaptive immunity, which supported by the ability to regulate cholesterol utilization in immune cells (14). However, the impact of TG and cholesterol levels on the immune effect of the hepatitis B vaccine has not been reported. Therefore, we discuss the potential impact of TG and cholesterol levels on the immune responses to the hepatitis B vaccine in this study.

## 2. Materials and methods

### 2.1. Study population

**Figure 1:**
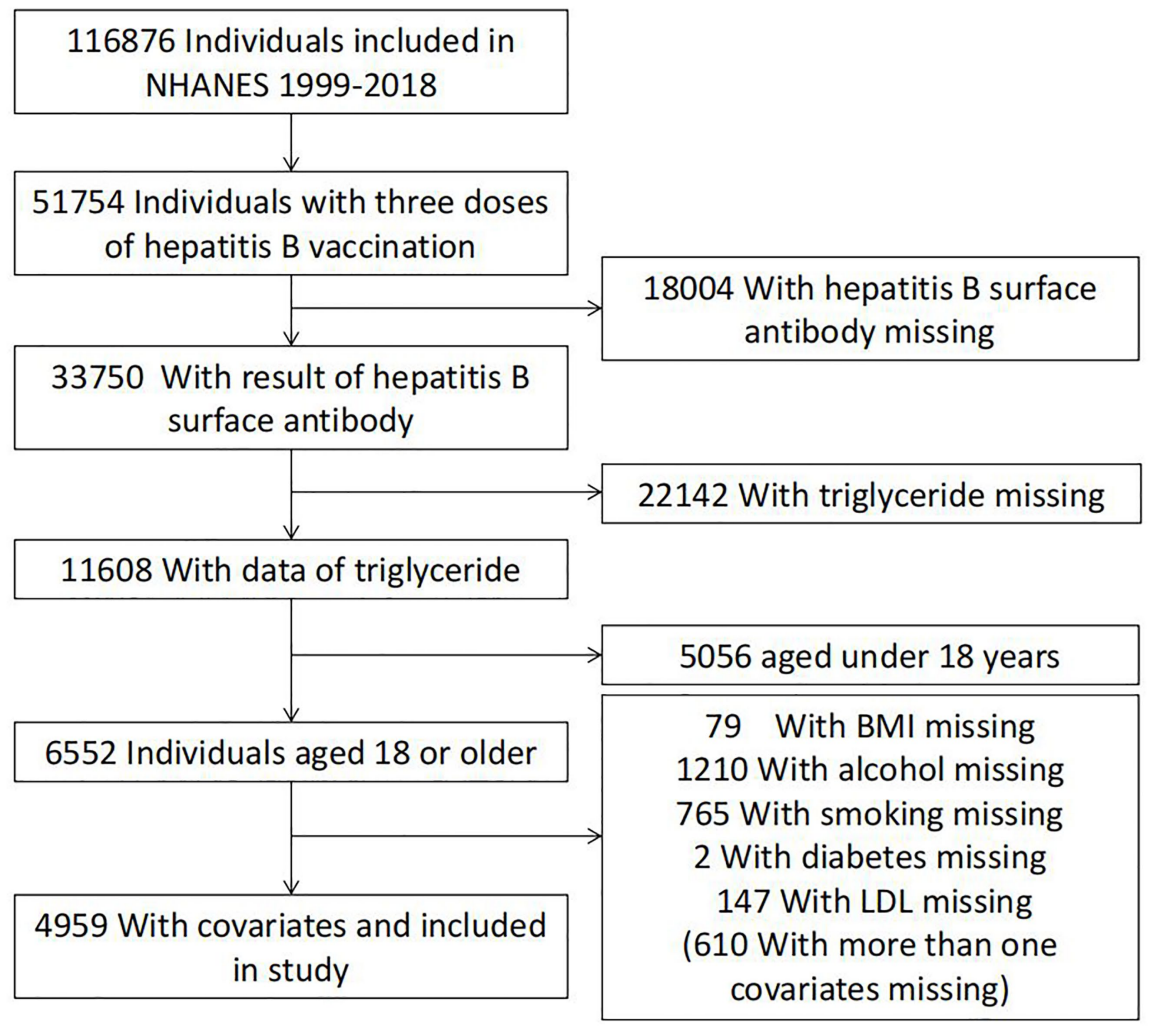
Flow chart of the population included in the final analysis of our study.

Guided by the National Center for Health Statistics (NCHS) of the Centers for Disease Control and Prevention (CDC), the National Health and Nutrition Examination Survey (NHANES) program investigates the health and nutritional status of people in the United States. NHANES data collection involves questionnaire surveys, laboratory analyses, and physical examinations. For this study, NHANES data collected between 1999 and 2018 were used. The samples were screened for the following inclusion criteria (to eliminate ineligible individuals): (I) individuals with full immunization with three doses of the hepatitis B vaccine, (II) individuals with hepatitis B virus surface antibody (anti-HBs) data, (III) individuals with total cholesterol (TC) values, triglyceride (TG) values, high-density lipoprotein (HDL) values, and low-density lipoprotein (LDL) values, and (IV) individuals aged ≥18 years. Overall, 4,959 individuals were included as shown in Figure 1.

### 2.2. Measurement of anti-HBs

Between 1999 and 2006, the levels of anti-HBs in the serum or plasma of individuals recruited for the NHANES program were detected with the AUSAB EIA kit with a solid-phase enzyme-linked immunoassay technique. The anti-HBs concentration was determined by comparison to a standard curve generated from repeated runs of standard measurements using the AUSAB EIA kit. After 2006, the concentration of anti-HBs was measured with an immunometric technique through the VITROS anti-HBs quantitative assay. The unit of anti-HBs was milli-international units per mL (mIU/mL). Laboratory protocols were obtained from the official NHANES website.^1^

### 2.3. Measurement of TG

From 1999 to 2006, TG were detected enzymatically using a series of coupled reactions. First, TGs were hydrolyzed to glycerol. Then, glycerol was oxidized to H_2_O_2_ by glycerol oxidase. After that, peroxidase was used to convert H2O2 to a phenazone. Finally, TG levels were determined by measuring the absorbance of phenazone at 500 nm. Between 2007 and 2012, a two-reagent, endpoint reaction was used to determine the TG concentration. First, a background absorbance value was obtained after a series of enzymatic reactions (free glycerol to glycerol-3-phosphate by glycerol kinase, then to hydrogen peroxide by glycerol phosphate oxidase, and then combined with 4-chlorophenol to an oxidation product by peroxidase) with reagent 1 (glycerol blanking). Then, driven by the reagents above, lipase was added to reagent 2 to convert TG to glycerol, and 4-aminophenzone was added to react with the hydrogen peroxide produced in the last reaction. The reaction was measured at 505 nm (secondary wavelength = 700 nm). After 2012, TG were hydrolyzed to glycerol using lipoprotein lipase from microorganisms and then oxidized to dihydroxyacetone phosphate and hydrogen peroxide. The hydrogen peroxide reacted with 4-aminobenzophenone and 4-chlorophenol under the catalysis of peroxidase to form a red dyestuff (Trinder endpoint reaction). The color intensity of the red dyestuff was proportional to the TG concentration and was measured photometrically. No adjustment of values was necessary to account for the change. Laboratory protocols were obtained from the official NHANES website.

### 2.4. Measurement of cholesterol

#### 2.4.1. Total cholesterol

From 1999 to 2006, total cholesterol was measured in serum or plasma using enzymes in a series of coupled reactions that hydrolyze cholesteryl esters and oxidize the 3-OH group of cholesterol. After 2007, Roche Modular P chemistry analyzer was used to detected total cholesterol level. No adjustment of values was necessary to account for the change. Detailed instructions showed in the official NHANES website.

#### 2.4.2. High-density lipoprotein cholesterol

The HDL level was using direct immunoassay method in serum. A detailed description of the laboratory method used can be found in Laboratory Procedures Manuals on the NHANES web site.

#### 2.4.3. Low-density lipoprotein cholesterol

The LDL concentration was estimated from total cholesterol value minus HDL and one fifth of the triglyceride value. The official NHANES website provides detailed instructions for the experiment.

### 2.5. Covariates

Based on the literature, some potential confounders have been confirmed to be related to the immune effect of the hepatitis B vaccine, including age, sex, race, body mass index (BMI), smoking status, drinking, and diabetes, which were controlled in this study. Age was categorized into three groups: 18–40 years old, 40–60 years old, and older than 60 years. Sex contains two types: males, females. Race was divided into five groups: Mexican American, other Hispanic, non-Hispanic white, non-Hispanic black, and other race (including multiracial). BMI was categorized as underweight (<18.5), normal weight (18.5–25), overweight (25–30), and obese (≥30) based on WHO classification criteria. Smoking status divided into two groups: never smoker (<100 cigarettes in life), smoker (>100 cigarettes in life). Drinking status divided into two groups: never drinker (<12 alcohol drinks every year), drinker (≥12 alcohol drinks every year). Diabetes status divided into two groups according to doctor’s diagnosis.

### 2.6. Statistical analysis

We performed statistical analysis by using the Solutions Statistical Package for the Social Sciences (IBM SPSS Statistics Premium V25.0). Continuous variables are shown as the means ±standard deviations (SDs), while categorical variables are shown as numbers and percentages (%) [N (%)]. The concentrations of TC, TG, HDL, and LDL were divided into the following groups, respectively: normal (≤5.69 mmol/l) and hyper (>5.69 mmol/l), normal (≤1.7 mmol/l) and hyper (>1.7 mmol/l), normal (≥0.90 mmol/l) and hypo (<0.90 mmol/l), and normal (≤3.36 mmol/l) and hyper (>3.36 mmol/l). For the baseline characteristics, if continuous variables conformed to a normal distribution or could be adjusted to a normal distribution after processing, such as *via* logarithmic transformation, Student’s *t-*test was used for comparisons between two groups; otherwise, the nonparametric test (Mann–Whitney *U* test) was performed (Supplementary materials showed detailed distribution). Categorical variables were compared between two groups using the chi-square test. We used two logistic regression models (crude model and adjusted model) to evaluate the relationship between the triglyceride and cholesterol and anti-HBs levels. Only TC, TG, HDL, and LDL were included in the crude model, while age, sex, race, BMI smoking status, drinking status, and diabetes status were adjusted in the adjusted model. Simultaneously, we conducted stratified analyses by age (18–40, 40–60, and >60 years), sex (male and female), BMI (<18.5, 18.5–25, 25–30, and ≥30), race (Mexican American, other Hispanic, non-Hispanic white, non-Hispanic black, and other races, including multiracial), smoking status (smoker and never smoker), drinking status (drinker and never drinker), and diabetes status (yes and no).

To diminish the selection bias among subgroups for age, sex, and race, the samples were weighted in the NHANES survey. Therefore, we chose the unweighted data in this study because the adjusted model included the variables used to count sample weights, as recommended previously (15, 16).

In this study, we used a two-tailed test of significance, with a *p-*value of <0.05 being considered statistically significant.

## 3. Results

### 3.1. Participant characteristics

The study included NHANES data collected from a total of 4,959 individuals between 1999 and 2018. The demographic characteristics are shown in Table 1, including age, sex, race, BMI, and TG concentration. A total of 2,304 subjects were positive for anti-HBs, while 2,655 subjects were negative. The average patient age was 39.19 ± 15.84 years. Significant differences in age, sex, race, BMI, smoking status, drinking status, TG, and HDL levels were discovered in the study population stratified by positivity for anti-HBs or not.

**Table 1:** Characteristics of 4,959 participants in NHANES data, 1999–2018.

Characteristics	Hepatitis B surface antibody is positive (*N* = 2,304)	Hepatitis B surface antibody is negative (*N* = 2,655)	*p*-value
Age (years)	37.51 ± 15.10	40.64 ± 16.32	<0.001^*,a^
18–40 years	1,423 (28.7%)	1,401 (28.3%)	<0.001^*^
40–60 years	606 (12.2%)	817 (16.5%)	
>60 years	275 (5.5%)	437 (8.8%)	
Sex			<0.001^*^
Males	870 (17.5%)	1,221 (24.6%)	
Females	1,434 (28.9%)	1,434 (28.9%)	
Race			<0.001^*^
Mexican American	236 (4.8%)	436 (8.8%)	
Other Hispanic	184 (3.7%)	252 (5.1%)	
Non-Hispanic white	1,044 (21.1%)	1,047 (21.1%)	
Non-Hispanic black	479 (9.7%)	660 (13.3%)	
Other race including multiracial	361 (7.3%)	260 (5.2%)	
BMI			<0.001^*^
Underweight	53 (1.1%)	47 (0.9%)	
Normal weight	864 (17.4%)	738 (14.9%)	
Overweight	697 (14.1%)	806 (16.3%)	
Obese	690 (13.9%)	1,064 (21.5%)	
Smoking status			<0.001^*^
Smoker	811 (16.4%)	1,086 (21.9%)	
Never smoker	1,493 (30.1%)	1,569 (31.6%)	
Drinking status			0.029^*^
Drinker	1,601 (32.3%)	1768 (35.7%)	
Never drinker	703 (14.2%)	887 (17.9%)	
Having diabetes or not			<0.001^*^
Yes	142 (2.9%)	305 (6.2%)	
No	2,162 (43.6%)	2,350 (47.4%)	
TC (mmol/L)	4.86 ± 1.04	4.85 ± 1.04	0.607^a^
Normal	1875 (37.8%)	2,156 (43.5%)	0.875
Hyper	429 (8.7%)	499 (10.1%)	
TG (mmol/L)	1.22 ± 0.75	1.33 ± 0.77	<0.001^*,a^
Normal	1853 (37.4%)	2020 (40.7%)	<0.001^*^
Hyper	451 (9.1%)	635 (12.8%)	
HDL (mmol/L)	1.45 ± 0.42	1.37 ± 0.39	<0.001^*,a^
Normal	2,198 (44.3%)	2,462 (49.6%)	<0.001^*^
Hypo	106 (2.1%)	193 (3.9%)	
LDL (mmol/L)	2.85 ± 0.90	2.87 ± 0.91	0.470^a^
Normal	1732 (34.9%)	1937 (39.1%)	0.076
Hyper	572 (11.5%)	718 (14.5%)	

PIR: family poverty income ratio. BMI: body mass index. TC: total cholesterol. TG: triglyceride. HDL: high-density lipoprotein. LDL: low-density lipoprotein. The data were presented as the means ± standard deviations (age, TC, TG, HDL, LDL) or N (%) (other indicators). *The p-value is less than 0.05. ^a^Calculated using Mann–Whitney *U* test.

### 3.2. Logistic regression model to assess the association between the triglyceride and cholesterol levels and anti-HBs results

We performed a logistic regression model to evaluate the effect of the triglyceride and cholesterol levels on the anti-HBs results. As shown in Table 2, regardless of the crude model or the adjusted model, higher HDL level showed a significant relationship with positive anti-HBs results (cOR = 1.479, 95% CI: 1.150, 1.901, *p* = 0.002; aOR = 1.304, 95% CI: 1.006, 1.691, *p* = 0.045). TG and LDL showed significant relationships in the crude model (cOR = 1.244, 95% CI: 1.077, 1.437, *p* = 0.003; cOR = 1.231, 95% CI: 1.029, 1.473, *p* = 0.023, respectively), while the adjusted model showed negative results.

**Table 2:** Odds ratios for associations between triglyceride and cholesterol levels and anti-HBs results.

	Crude model		Adjusted model	
	cOR (95% CI)	*P-*value	aOR (95% CI)	*P-*value
TC		0.062		0.305
Normal	Ref		Ref	
Hyper	0.822 (0.669, 1.010)		0.895 (0.724, 1.106)	
TG		0.003		0.474
Normal	Ref		Ref	
Hyper	1.244 (1.077, 1.437)		1.058 (0.907, 1.233)	
HDL		0.002		
Normal	Ref		Ref	0.045
Hypo	1.479 (1.150, 1.901)		1.304 (1.006, 1.691)	
LDL		0.023		0.317
Normal	Ref		Ref	
Hyper	1.231 (1.029, 1.473)		1.099 (0.913, 1.324)	

The crude model only included total cholesterol, triglyceride, high-density lipoprotein, and low-density lipoprotein; the adjusted model comprised the following covariates: age, sex, race, BMI, smoking status, drinking status, and diabetes.

### 3.3. Logistic regression model to assess the association between the triglyceride and cholesterol levels and anti-HBs results stratified by age, sex, race, BMI, smoking status, drinking status, and diabetes

Several factors were very different between positive and negative anti-HBs results according to Table 1. To assess the effect of the results, we performed group analyses of these factors separately. The relationships between the triglyceride and cholesterol levels and anti-HBs results stratified by age, sex, race, BMI, smoking status, drinking status, and diabetes are shown in Tables 3–9, respectively.

**Table 3:** Odds ratios for associations between triglyceride and cholesterol levels and anti-HBs results stratified by age.

Age	TC (Hyper)				TG (Hyper)			
	Crude model		Adjusted model		Crude model		Adjusted model	
	cOR (95% CI)	*P-*value	aOR (95% CI)	*P-*value	cOR (95% CI)	*P-*value	aOR (95% CI)	*P-*value
18–40 years		0.163		0.403		0.456		0.862
Normal	Ref		Ref		Ref		Ref	
Hyper/Hypo	0.807 (0.597, 1.090)		0.877 (0.644, 1.194)		1.083 (0.879, 1.334)		0.981 (0.788, 1.221)	
40–60 years		0.235		0.597		0.005		0.240
Normal	Ref		Ref		Ref		Ref	
Hyper/Hypo	0.814 (0.581, 1.143)		0.910 (0.641, 1.292)		1.435 (1.113, 1.850)		1.177 (0.896, 1.547)	
>60 years		0.616		0.772		0.745		0.843
Normal	Ref		Ref		Ref		Ref	
Hyper/Hypo	0.871 (0.508, 1.495)		1.086 (0.621, 1.899)		1.060 (0.748, 1.502)		0.964 (0.668, 1.390)	
Age	HDL (Hypo)				LDL (Hyper)			
	Crude model		Adjusted model		Crude model		Adjusted model	
	cOR (95% CI)	*P-*value	aOR (95% CI)	*P-*value	cOR (95% CI)	*P-*value	aOR (95% CI)	*P-*value
18–40 years		0.002		0.024		0.005		0.022
Normal	Ref		Ref		Ref		Ref	
Hyper/Hypo	1.643 (1.198, 2.253)		1.455 (1.050, 2.017)		1.421 (1.109, 1.820)		1.347 (1.043, 1.740)	
40–60 years		0.681		0.606		0.933		0.456
Normal	Ref		Ref		Ref		Ref	
Hyper/Hypo	1.107 (0.682, 1.798)		0.876 (0.529, 1.450)		0.987 (0.723, 1.346)		0.884 (0.639, 1.223)	
>60 years		0.044		0.078		0.846		0.601
Normal	Ref		Ref		Ref		Ref	
Hyper/Hypo	2.572 (1.027, 6.441)		2.312 (0.909, 5.881)		0.952 (0.579, 1.565)		0.872 (0.522, 1.457)	

The crude model only included total cholesterol, triglyceride, high-density lipoprotein, and low-density lipoprotein; the adjusted model comprised the following covariates: sex, race, BMI, smoking status, drinking status, and diabetes status.

**Table 4:** Odds ratios for associations between triglyceride and cholesterol levels and anti-HBs results stratified by sex.

Sex	TC (Hyper)				TG (Hyper)			
	Crude model		Adjusted model		Crude model		Adjusted model	
	cOR (95% CI)	*P-*value	aOR (95% CI)	*P-*value	cOR (95% CI)	*P-*value	aOR (95% CI)	*P-*value
Male		0.562		0.414		0.373		0.824
Normal	Ref		Ref		Ref		Ref	
Hyper/Hypo	0.909 (0.659, 1.254)		0.872 (0.627, 1.212)		1.101 (0.891, 1.361)		1.026 (0.820, 1.282)	
Female		0.182		0.552		0.006		0.489
Normal	Ref		Ref		Ref		Ref	
Hyper/Hypo	0.831 (0.633, 1.091)		0.919 (0.694, 1.215)		1.319 (1.082, 1.608)		1.078 (0.872, 1.332)	
Sex	HDL (Hypo)				LDL (Hyper)			
	Crude model		Adjusted model		Crude model		Adjusted model	
	cOR (95% CI)	*P-*value	aOR (95% CI)	*P-*value	cOR (95% CI)	*P-*value	aOR (95% CI)	*P-*value
Male		0.177		0.133		0.492		0.568
Normal	Ref		Ref		Ref		Ref	
Hyper/Hypo	1.231 (0.911, 1.663)		1.269 (0.930, 1.732)		1.097 (0.843, 1.426)		1.082 (0.825, 1.419)	
Female		0.013		0.045		0.069		0.445
Normal	Ref		Ref		Ref		Ref	
Hyper/Hypo	1.832 (1.137, 2.950)		1.646 (1.011, 2.678)		1.259 (0.982, 1.613)		1.105 (0.855, 1.430)	

The crude model only included total cholesterol, triglyceride, high-density lipoprotein, and low-density lipoprotein; the adjusted model comprised the following covariates: age, race, BMI, smoking status, drinking status, and diabetes status.

**Table 5:** Odds ratios for associations between triglyceride and cholesterol levels and anti-HBs results stratified by race.

Race	TC (Hyper)				TG (Hyper)			
	Crude model		Adjusted model		Crude model		Adjusted model	
	cOR (95% CI)	*P-*value	aOR (95% CI)	*P-*value	cOR (95% CI)	*P-*value	aOR (95% CI)	*P-*value
Mexican American		0.380		0.153		0.830		0.223
Normal	Ref		Ref		Ref		Ref	
Hyper/Hypo	1.296 (0.726, 2.313)		1.546 (0.850, 2.813)		1.041 (0.721, 1.503)		0.781 (0.524, 1.163)	
Other Hispanic		0.879		0.977		0.505		0.336
Normal	Ref		Ref		Ref		Ref	
Hyper/Hypo	0.947 (0.470, 1.909)		0.989 (0.478, 2.049)		0.846 (0.518, 1.383)		0.776 (0.463, 1.301)	
Non-Hispanic white		0.002		0.012		< 0.001		0.101
Normal	Ref		Ref		Ref		Ref	
Hyper/Hypo	0.611 (0.445, 0.839)		0.658 (0.475, 0.911)		1.490 (1.205, 1.842)		1.206 (0.964, 1.509)	
Non-Hispanic black		0.641		0.796		0.661		0.774
Normal	Ref		Ref		Ref		Ref	
Hyper/Hypo	0.897 (0.568, 1.416)		0.940 (0.590, 1.499)		1.097 (0.726, 1.656)		0.940 (0.616, 1.435)	
Other race including multiracial		0.198		0.162		0.101		0.393
Normal	Ref		Ref		Ref		Ref	
Hyper/Hypo	1.460 (0.820, 2.600)		1.526 (0.844, 2.758)		1.400 (0.936, 2.094)		1.203 (0.788, 1.837)	
Race	HDL (Hypo)				LDL (Hyper)			
	Crude model		Adjusted model		Crude model		Adjusted model	
	cOR (95% CI)	*P-*value	aOR (95% CI)	*P-*value	cOR (95% CI)	*P-*value	aOR (95% CI)	*P-*value
Mexican American		0.071		0.159		0.944		0.366
Normal	Ref		Ref		Ref		Ref	
Hyper/Hypo	1.877 (0.947, 3.718)		1.674 (0.817, 3.428)		1.018 (0.621, 1.668)		0.786 (0.467, 1.324)	
Other Hispanic		0.110		0.195		0.172		0.646
Normal	Ref		Ref		Ref		Ref	
Hyper/Hypo	1.979 (0.856, 4.576)		1.764 (0.748, 4.162)		1.515 (0.835, 2.748)		1.157 (0.620, 2.161)	
Non-Hispanic white		0.027		0.140		0.108		0.308
Normal	Ref		Ref		Ref		Ref	
Hyper/Hypo	1.487 (1.046, 2.115)		1.316 (0.913, 1.896)		1.262 (0.951, 1.675)		1.164 (0.869, 1.559)	
Non-Hispanic black		0.246		0.515		0.149		0.293
Normal	Ref		Ref		Ref		Ref	
Hyper/Hypo	1.537 (0.743, 3.181)		1.279 (0.610, 2.681)		1.326 (0.904, 1.945)		1.235 (0.833, 1.832)	
Other race including multiracial		0.602		0.451		0.741		0.538
Normal	Ref		Ref		Ref		Ref	
Hyper/Hypo	0.810 (0.366, 1.790)		0.730 (0.322, 1.654)		0.918 (0.551, 1.529)		0.847 (0.500, 1.435)	

The crude model only included total cholesterol, triglyceride, high-density lipoprotein, and low-density lipoprotein; the adjusted model comprised the following covariates: age, sex, BMI, smoking status, drinking status, and diabetes status.

**Table 6:** Odds ratios for associations between triglyceride and cholesterol levels and anti-HBs results stratified by BMI.

BMI	TC (Hyper)				TG (Hyper)			
	Crude model		Adjusted model		Crude model		Adjusted model	
	cOR (95% CI)	*P-*value	aOR (95% CI)	*P-*value	cOR (95% CI)	*P-*value	aOR (95% CI)	*P-*value
Underweight		0.804		0.859		0.422		0.376
Normal	Ref		Ref		Ref		Ref	
Hyper/Hypo	1.545 (0.050, 47.521)		1.431 (0.027, 75.413)		3.336 (0.176, 63.369)		4.215 (0.174, 102.082)	
Normal weight		0.477		0.957		0.007		0.062
Normal	Ref		Ref		Ref		Ref	
Hyper/Hypo	0.862 (0.573, 1.297)		1.012 (0.661, 1.549)		1.566 (1.129, 2.173)		1.385 (0.984, 1.950)	
Overweight		0.713		0.776		0.416		0.645
Normal	Ref		Ref		Ref		Ref	
Hyper/Hypo	1.069 (0.751, 1.521)		1.05 (0.733, 1.515)		1.107 (0.866, 1.416)		1.062 (0.822, 1.372)	
Obese		0.031		0.073		0.989		0.470
Normal	Ref		Ref		Ref		Ref	
Hyper/Hypo	0.693 (0.497, 0.966)		0.733 (0.522, 1.030)		1.002 (0.802, 1.251)		0.917 (0.724, 1.160)	
BMI	HDL (Hypo)				LDL (Hyper)			
	Crude model		Adjusted model		Crude model		Adjusted model	
	cOR (95% CI)	*P-*value	aOR (95% CI)	*P-*value	cOR (95% CI)	*P-*value	aOR (95% CI)	*P-*value
Underweight		0.833		0.956		0.995		0.882
Normal	Ref		Ref		Ref		Ref	
Hyper/Hypo	0.659 (0.014, 31.757)		0.895 (0.018, 44.839)		1.010 (0.045, 22.896)		1.294 (0.043, 39.018)	
Normal weight		0.247		0.780		0.617		0.888
Normal	Ref		Ref		Ref		Ref	
Hyper/Hypo	1.511 (0.751, 3.040)		1.108 (0.541, 2.269)		1.098 (0.761, 1.584)		0.973 (0.666, 1.421)	
Overweight		0.200		0.186		0.653		0.676
Normal	Ref		Ref		Ref		Ref	
Hyper/Hypo	1.326 (0.861, 2.042)		1.349 (0.865, 2.103)		0.931 (0.681, 1.272)		0.934 (0.678, 1.287)	
Obese		0.055		0.055		0.032		0.040
Normal	Ref		Ref		Ref		Ref	
Hyper/Hypo	1.413 (0.993, 2.012)		1.432 (0.992, 2.068)		1.371 (1.027, 1.829)		1.364 (1.014, 1.833)	

The crude model only included total cholesterol, triglyceride, high-density lipoprotein, and low-density lipoprotein; the adjusted model comprised the following covariates: age, sex, race, smoking status, drinking status, and diabetes status.

**Table 7:** Odds ratios for associations between triglyceride and cholesterol levels and anti-HBs results stratified by smoking status.

Smoking	TC (Hyper)				TG (Hyper)			
	Crude model		Adjusted model		Crude model		Adjusted model	
	cOR (95% CI)	*P-*value	aOR (95% CI)	*P-*value	cOR (95% CI)	*P-*value	aOR (95% CI)	*P-*value
Smoker		0.529		0.738		0.231		0.416
Normal	Ref		Ref		Ref		Ref	
Hyper/Hypo	0.900 (0.649,1.249)		0.944 (0.675, 1.320)		1.141 (0.919, 1.416)		1.098 (0.876, 1.377)	
Never smoker		0.042		0.351		0.013		0.726
Normal	Ref		Ref		Ref		Ref	
Hyper/Hypo	0.759 (0.582, 0.990)		0.877 (0.665, 1.156)		1.282 (1.054, 1.559)		1.038 (0.842, 1.281)	
Smoking	HDL (Hypo)				LDL (Hyper)			
	Crude model		Adjusted model		Crude model		Adjusted model	
	cOR (95% CI)	*P-*value	aOR (95% CI)	*P-*value	cOR (95% CI)	*P-*value	aOR (95% CI)	*P-*value
Smoker		0.005		0.012		0.170		0.396
Normal	Ref		Ref		Ref		Ref	
Hyper/Hypo	1.671 (1.164, 2.400)		1.608 (1.111, 2.327)		1.228 (0.915, 1.648)		1.140 (0.843, 1.541)	
Never smoker		0.216		0.866		0.061		0.702
Normal	Ref		Ref		Ref		Ref	
Hyper/Hypo	1.250 (0.878, 1.780)		1.033 (0.712, 1.498)		1.243 (0.990, 1.560)		1.048 (0.825, 1.330)	

The crude model only included total cholesterol, triglyceride, high-density lipoprotein, and low-density lipoprotein; the adjusted model comprised the following covariates: age, sex, race, BMI, drinking status, and diabetes status.

**Table 8:** Odds ratios for associations between triglyceride and cholesterol levels and anti-HBs results stratified by drinking status.

Drinking status	TC (Hyper)				TG (Hyper)			
	Crude model		Adjusted model		Crude model		Adjusted model	
	cOR (95% CI)	*P-*value	aOR (95% CI)	*P-*value	cOR (95% CI)	*P-*value	aOR (95% CI)	*P-*value
Drinker		0.031		0.148		0.015		0.584
Normal	Ref		Ref		Ref		Ref	
Hyper/Hypo	0.764 (0.598, 0.976)		0.829 (0.643, 1.069)		1.242 (1.043, 1.479)		1.053 (0.875, 1.267)	
Never drinker		0.964		0.769		0.103		0.819
Normal	Ref		Ref		Ref		Ref	
Hyper/Hypo	0.991 (0.678, 1.450)		1.060 (0.718, 1.566)		1.238 (0.958, 1.600)		1.033 (0.785, 1.359)	
Drinking	HDL (Hypo)				LDL (Hyper)			
	Crude model		Adjusted model		Crude model		Adjusted model	
	cOR (95% CI)	*P-*value	aOR (95% CI)	*P-*value	cOR (95% CI)	*P-*value	aOR (95% CI)	*P-*value
Drinker		0.003		0.045		0.002		0.055
Normal	Ref		Ref		Ref		Ref	
Hyper/Hypo	1.560 (1.162, 2.096)		1.367 (1.007, 1.855)		1.414 (1.141, 1.754)		1.245 (0.995, 1.558)	
Never drinker		0.288		0.617		0.517		0.238
Normal	Ref		Ref		Ref		Ref	
Hyper/Hypo	1.297 (0.803, 2.095)		1.136 (0.690, 1.869)		0.897 (0.646, 1.246)		0.815 (0.581, 1.144)	

The crude model only included total cholesterol, triglyceride, high-density lipoprotein, and low-density lipoprotein; the adjusted model comprised the following covariates: age, sex, race, BMI, smoking status, and diabetes status.

**Table 9:** Odds ratios for associations between triglyceride and cholesterol levels and anti-HBs results stratified by diabetes status.

Diabetes status	TC (Hyper)				TG (Hyper)			
	Crude model		Adjusted model		Crude model		Adjusted model	
	cOR (95% CI)	*P-*value	aOR (95% CI)	*P-*value	cOR (95% CI)	*P-*value	aOR (95% CI)	*P-*value
Yes		0.619		0.714		0.848		0.375
Normal	Ref		Ref		Ref		Ref	
Hyper/Hypo	0.791 (0.315, 1.990)		0.838 (0.324, 2.164)		0.959 (0.623, 1.476)		0.808 (0.504, 1.295)	
No		0.072		0.289		0.014		0.385
Normal	Ref		Ref		Ref		Ref	
Hyper/Hypo	0.823 (0.666, 1.017)		0.889 (0.715, 1.105)		1.213 (1.039, 1.417)		1.075 (0.913, 1.265)	
Diabetes	HDL (Hypo)				LDL (Hyper)			
	Crude model		Adjusted model		Crude model		Adjusted model	
	cOR (95% CI)	*P-* value	aOR (95% CI)	*P-*value	cOR (95% CI)	*P-*value	aOR (95% CI)	*P-*value
Yes		0.098		0.155		0.315		0.336
Normal	Ref		Ref		Ref		Ref	
Hyper/Hypo	2.192 (0.865, 5.554)		2.015 (0.767, 5.292)		1.543 (0.662, 3.596)		1.540 (0.639, 3.712)	
No		0.006		0.084		0.022		0.413
Normal	Ref		Ref		Ref		Ref	
Hyper/Hypo	1.446 (1.112, 1.881)		1.270 (0.968, 1.666)		1.239 (1.031, 1.490)		1.083 (0.895, 1.311)	

The crude model only included total cholesterol, triglyceride, high-density lipoprotein, and low-density lipoprotein; the adjusted model comprised the following covariates: age, sex, race, BMI, smoking status, and drinking status.

As shown in Table 3, the association between hypo-HDL and hyper-LDL and negative anti-HBs results was significant among the 18- to 40-year-old group in the crude model (cOR = 1.643, 95% CI: 1.198, 2.253, *p* = 0.002; cOR = 1.421, 95% CI: 1.109, 1.820, *p* = 0.005, respectively), while after further adjustment for other covariates, the result was still statistically significant (aOR = 1.455, 95% CI: 1.050, 2.017, *p* = 0.024; aOR = 1.347, 95% CI: 1.043, 1.740, *p* = 0.022, respectively). We discovered no relationship between TC and TG and negative anti-HBs results among all age groups, as well as HDL and LDL in 40- to 60- year-old group and the older than 60 years group.

The relationships between the triglyceride and cholesterol levels and negative anti-HBs results stratified by sex are displayed in Table 4. We found a significant relationship between the TG and negative anti-HBs results in females (cOR = 1.319, 95% CI: 1.082, 1.608, *p* = 0.006) in the crude model, while after adjusting for all covariates, the difference was no longer statistically significant. HDL showed a positive relationship with negative anti-HBs results both in the crude model and adjusted model (cOR = 1.832, 95% CI: 1.137, 2.950, *p* = 0.013; aOR = 1.646, 95% CI: 1.011, 2.678, *p* = 0.045).

The results stratified by race are shown in Table 5. In the non-Hispanic white group, TC were positively associated with a negative anti-HBs result (cOR = 0.611, 95% CI: 0.445, 0.839, *p* = 0.002; aOR = 0.658, 95% CI: 0.475, 0.911, *p* = 0.012, respectively). Positive relationships about TG and HDL and anti-HBs result also appeared in the non-Hispanic white group in the crude model (cOR = 1.490, 95% CI: 1.205, 1.842, *p* < 0.001; cOR = 1.487, 95% CI: 1.046, 2.115, *p* = 0.027), while the results showed negative in the adjusted model.

We performed the results stratified by BMI in Table 6. In the obese group, whether in the crude model or in the adjusted model, the hyper-LDL level was a risk factor for a negative anti-HBs result (cOR = 1.371, 95% CI: 1.027, 1.829, *p* = 0.032; aOR = 1.364, 95% CI: 1.014, 1.833, *p* = 0.040). TC only showed significant result in the obese group in the crude model rather than the adjusted model. In the underweight group, the normal-weight group, and the overweight group, however, the results showed no statistical significance.

Table 7 showed the results stratified by smoking status. In the never smoking group, TC and TG showed a positive relationship with anti-HBs results only in the crude model. At the same time, we found a significant relationship between the HDL and negative anti-HBs results in the smoker group both in the crude model and in the adjusted model (cOR = 1.671, 95% CI: 1.164, 2.400, *p* = 0.005; aOR = 1.608, 95% CI: 1.111, 2.327, *p* = 0.012).

The results classified by drinking status are shown in Table 8. In the crude model, the TC, TG, HDL, and LDL were all positively associated with a negative anti-HBs result in the drinker group. However, after adjusting for all covariates, the difference was only existed in the relationship between the HDL level and anti-HBs result (aOR = 1.367, 95% CI: 1.007, 1.855, *p* = 0.012). In the never drinker group, the results showed no statistical significance.

At last, we performed subgroup analyses divided by diabetes status (Table 9). In the no diabetes group, the results discovered the TG, HDL, and LDL all had positive relation with a negative anti-HBs result, while the adjusted model showed negative results.

## 4. Discussion

This was a cross-sectional study to assess the relationship between the TG and cholesterol levels and negative anti-HBs result among 4,959 individuals aged 18 years and older in the United States. Our results demonstrated that higher TC level was showed a positive relationship with a positive anti-HBs result among participants who were Non-Hispanic white; HDL level was associated with a negative anti-HBs result among participants aged 18- to 40-year-old, participants who were female, participants who were smoking more than 100 cigarettes in life, and drinking more than 12 times every year; LDL level was associated with a negative anti-HBs result among participants aged 18- to 40-year-old, and participants who were obese. Generally, we found that the cholesterol level was related to a negative anti-HBs result, rather than hypertriglyceridemia. High level of HDL and low level of LDL may be protective factors of better immune effect of hepatitis B vaccine.

Recognized as the most effective and affordable tool to prevent HBV infection, the hepatitis B vaccine has been widely used since its launch in 1982, resulting in dramatic reductions in the HBV carrier rate as well as hepatitis B-related morbidity and mortality (3, 17). Despite great achievements, there are still some challenges associated with hepatitis B immunization. One of the crucial challenges is nonresponse or low response to the hepatitis B vaccine. This may be due to the types of vaccines, doses, procedures and other attributes of vaccination but may also be due to certain recipient factors. Studies have shown that older age, obesity, genetic factors, etc., can affect the immune effect of the hepatitis B vaccine (18, 19). To date, there have been few related reports on the influence of TG or cholesterol levels. Our study found that the cholesterol level may affect individuals aged 18–40 years rather than elderly individuals. A systematic review of 30 years of experience by Ende C and colleagues indicated that people aged 60 years and older are less likely to exhibit a seroprotective response to the hepatitis B vaccine (5). Hence, other effects of old age, such as degeneration of immune function, are more prominent in elderly individuals, while the effect of the cholesterol level is more pronounced in relatively young recipients. Liu et al. discovered that people with BMI more than 25 kg/m^2^ compared to less than 25 kg/m^2^ were less prone to respond to the hepatitis B vaccine, and the risk of non-responsiveness among obese people increased with BMI (20). Our study showed that the LDL level was more conspicuous in obese subjects than in normal-weight subjects. We expected that the effect of obesity would be more pronounced than that of LDL. Interestingly, we found higher TC level was showed a positive relationship with a positive anti-HBs result among participants who were Non-Hispanic white. Differences in different ethnic groups may also be caused by the confounding of genetic factors. According to previous studies, smoking and drinking may be potential adverse factors in the immune effect of hepatitis B vaccine. Our results showed that the protective effect of HDL was more pronounced in the smoking and drinking groups.

At present, there is basically no published research on the immune responses of TG and cholesterol on the hepatitis B vaccine. Previous studies have focused more on the effect of obesity (20, 21). They found that obesity was significantly related to a nonresponse to the hepatitis B vaccine, potentially due to mechanisms such as leptin-induced systemic and B-cell intrinsic inflammation (22, 23), impaired T-cell responses (24), oxidative stress, and chronic low-grade inflammation (25, 26). High blood cholesterol and triglyceride are common comorbidities in obesity (27). The prevalence of hypertriglyceridemia in adults is approximately 10 % with considerable geographical differences (28–31). The effect of TG and cholesterol on vaccine immune responses has not yet been reported. As metabolic diseases, such as obesity, with some similar pathogenic mechanisms, the relationship between hypertriglyceridemia and cholesterol disorder and the immune responses to the hepatitis B vaccine was to be explored. Cholesterol homeostasis is critical for the maintenance of cellular and body activities, and is controlled by a series of rigorous regulatory mechanisms (32). Besides, the cholesterol signal pathway plays a role in the immune response through sterol response element-binding protein (SREBP) (13). HDL cholesterol, traditionally been considered the “good cholesterol” as high plasma levels are strongly associated with a lower risk of atherosclerotic cardiovascular disease (ASCVD) (33), may also play an important role in innate and adaptive immunity supported by the ability to regulate the availability of cholesterol in immune cells. We speculate that these underlying mechanisms may play a role in the process of cholesterol affecting the immune effect of hepatitis B vaccine, of course, this needs to be verified by more and more in-depth studies in the future.

Our research had several limitations. First, the limitation of a cross-sectional study prevented us from definitively identifying causal relationships. In addition, although we adjusted for some potential confounding factors, limited by the source of the original data, we were unable to obtain data on factors such as vaccine variety and dose, familial hypercholesterolemia and hypertriglyceridemia, etc., which may be important unadjusted confounding factors that could affect the results of this study. Moreover, the time interval between anti-HBs assessment and hepatitis B vaccination was unknown. Because anti-HBs concentrations gradually decline over time, this suggests that the two groups of individuals included are not so comparable, which may affect the results to some extent. More research should be conducted to investigate the influence of the cholesterol level on the immune responses to the hepatitis B vaccine, and more in-depth research should be performed to uncover the mechanism.

## Data availability statement

The original contributions presented in the study are included in the article/Supplementary material, further inquiries can be directed to the corresponding author.

## Ethics statement

The studies involving human participants were reviewed and approved by the Ethics Committee of the Affiliated Jiangning Hospital of Nanjing Medical University. The patients/participants provided their written informed consent to participate in this study. Written informed consent was obtained from the individual(s) for the publication of any potentially identifiable images or data included in this article.

## Author contributions

DG formulated the idea for this study and wrote the first draft of the manuscript. DG, JD, and RJ performed the analyses. QZ, NW, CW, HT, HJ, CZ, JM, and JX participated in sample collection and critically reviewed the manuscript. All authors contributed to the article and approved the submitted version.

## Funding

This work was supported by the Chinese Foundation for Hepatitis Prevention and Control, No. YGFK20190041.

## Conflict of interest

The authors declare that the research was conducted in the absence of any commercial or financial relationships that could be construed as a potential conflict of interest.

## Publisher’s note

All claims expressed in this article are solely those of the authors and do not necessarily represent those of their affiliated organizations, or those of the publisher, the editors and the reviewers. Any product that may be evaluated in this article, or claim that may be made by its manufacturer, is not guaranteed or endorsed by the publisher.

## Footnotes

^1^ https://wwwn.cdc.gov/nchs/data/nhanes/1999-2018/labmethods/pfc_g_met.pdf
