# Supplementary material for: The relationship between triglyceride, cholesterol and lipoprotein levels, and immune responses to hepatitis B vaccine
Source: Front Med (Lausanne). 2023 Mar 30;10:1131373. doi: 10.3389/fmed.2023.1131373 (PMC10098103; doi:10.3389/fmed.2023.1131373)

Supplementary Table1. Distribution table of age, total cholesterol, triglyceride, HDL-cholesterol, and LDL-cholesterol.

|  |  | Age | Total cholesterol | Triglyceride | HDL-cholesterol | LDL-cholesterol |
| --- | --- | --- | --- | --- | --- | --- |
| N | Valid | 4959 | 4959 | 4959 | 4959 | 4959 |
|  | Missing | 0 | 0 | 0 | 0 | 0 |
| Skewness | | 0.621 | 0.683 | 1.074 | 1.468 | 0.692 |
| Std. Error of Skewness | | 0.035 | 0.035 | 0.035 | 0.035 | 0.035 |
| Kurtosis | | -0.609 | 1.329 | 2.708 | 2.334 | 1.506 |
| Std. Error of Kurtosis | | 0.070 | 0.070 | 0.070 | 0.070 | 0.070 |

Supplementary Figure1. Histogram of age distribution. A. Total participants; B. Group with positive anti-HBs; C. Group with negative anti-HBs.

Supplementary Figure2. Histogram of total cholesterol distribution.


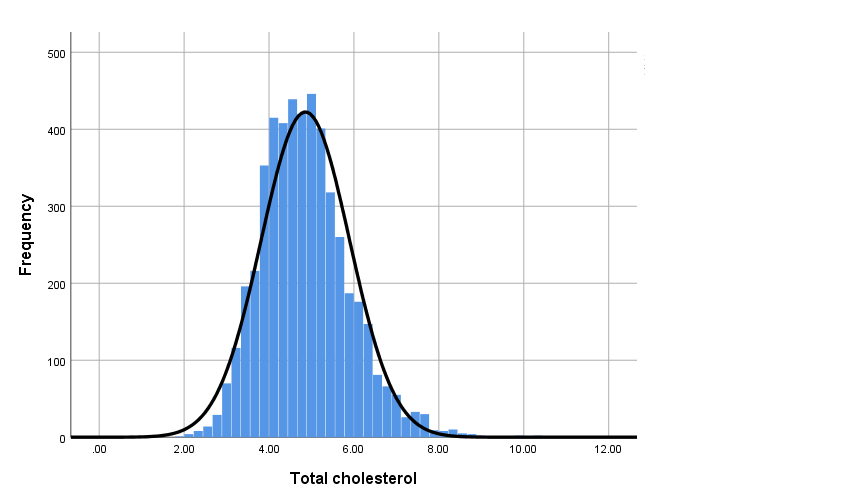


Supplementary Figure3. Histogram of triglyceride distribution.


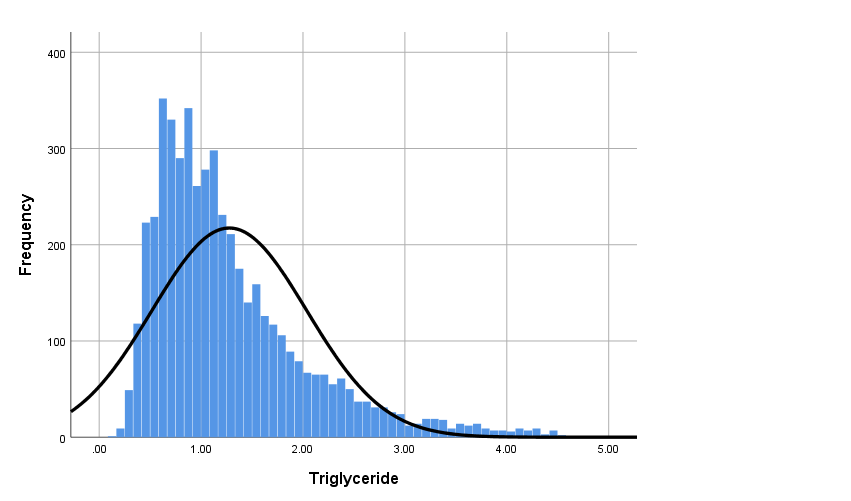


Supplementary Figure4. Histogram of HDL-cholesterol distribution.


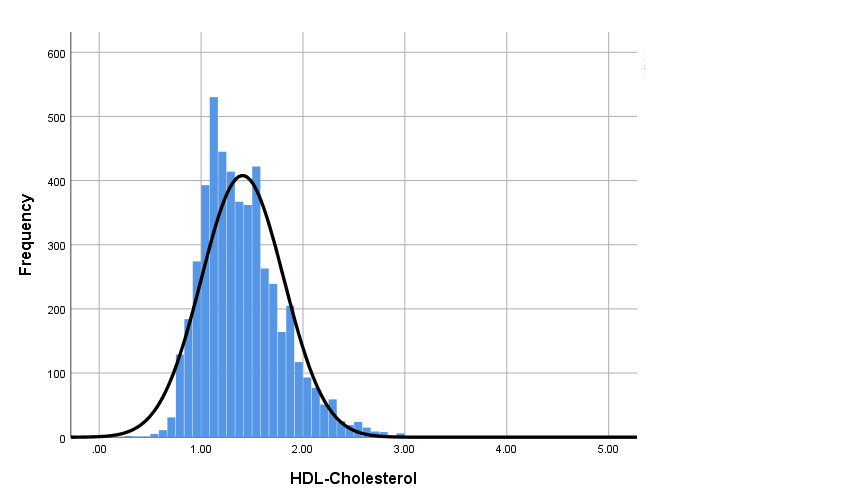


Supplementary Figure5. Histogram of LDL-cholesterol distribution.


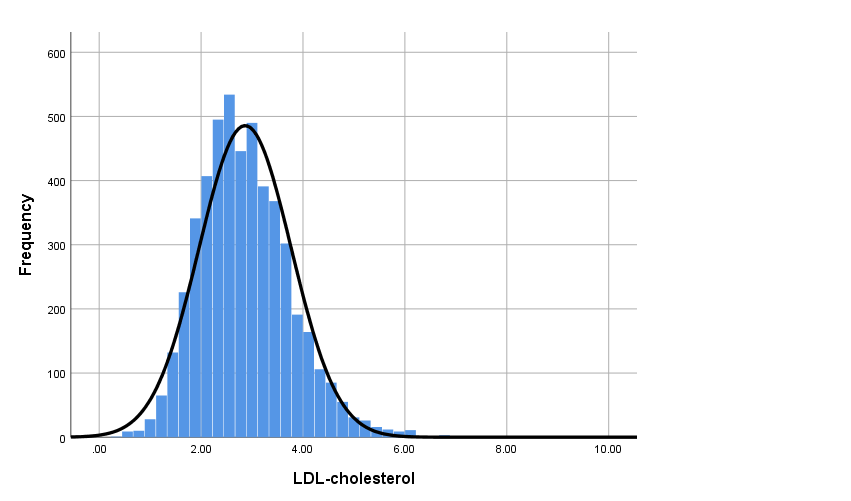

Supplement: Supplementary file 1 [file Data_Sheet_1.docx]
